# Lessons from relatives: C_4_ photosynthesis enhances CO_2_ assimilation during the low-light phase of fluctuations

**DOI:** 10.1093/plphys/kiad355

**Published:** 2023-06-19

**Authors:** Lucίa Arce Cubas, Cristina Rodrigues Gabriel Sales, Richard L Vath, Emmanuel L Bernardo, Angela C Burnett, Johannes Kromdijk

**Affiliations:** Department of Plant Sciences, University of Cambridge, Downing Street, CB2 3EA Cambridge, UK; Department of Plant Sciences, University of Cambridge, Downing Street, CB2 3EA Cambridge, UK; Department of Plant Sciences, University of Cambridge, Downing Street, CB2 3EA Cambridge, UK; Department of Plant Sciences, University of Cambridge, Downing Street, CB2 3EA Cambridge, UK; Institute of Crop Science, College of Agriculture and Food Science, University of the Philippines Los Baños, College, Laguna 4031, Philippines; Department of Plant Sciences, University of Cambridge, Downing Street, CB2 3EA Cambridge, UK; Department of Plant Sciences, University of Cambridge, Downing Street, CB2 3EA Cambridge, UK; Carl R Woese Institute for Genomic Biology, University of Illinois at Urbana-Champaign, Urbana, IL 61801, USA

## Abstract

Despite the global importance of species with C_4_ photosynthesis, there is a lack of consensus regarding C_4_ performance under fluctuating light. Contrasting hypotheses and experimental evidence suggest that C_4_ photosynthesis is either less or more efficient in fixing carbon under fluctuating light than the ancestral C_3_ form. Two main issues have been identified that may underly the lack of consensus: neglect of evolutionary distance between selected C_3_ and C_4_ species and use of contrasting fluctuating light treatments. To circumvent these issues, we measured photosynthetic responses to fluctuating light across 3 independent phylogenetically controlled comparisons between C_3_ and C_4_ species from *Alloteropsis*, *Flaveria*, and *Cleome* genera under 21% and 2% O_2_. Leaves were subjected to repetitive stepwise changes in light intensity (800 and 100 *µ*mol m^−2^ s^−1^ photon flux density) with 3 contrasting durations: 6, 30, and 300 s. These experiments reconciled the opposing results found across previous studies and showed that (i) stimulation of CO_2_ assimilation in C_4_ species during the low-light phase was both stronger and more sustained than in C_3_ species; (ii) CO_2_ assimilation patterns during the high-light phase could be attributable to species or C_4_ subtype differences rather than photosynthetic pathway; and (iii) the duration of each light step in the fluctuation regime can strongly influence experimental outcomes.

## Teaser

Comparing photosynthesis in 3 pairs of closely related C_3_ and C_4_ species across 3 fluctuating light regimes shows that C_4_ photosynthesis has a systematic advantage under the low-light phase.

## Introduction

Plants exhibiting C_4_ photosynthesis are mostly found in warm, high-light environments (Sage 2000). Although these environments have high-light intensity at the top of the canopy, the light conditions experienced by leaves within the canopy can be extremely dynamic. Indeed, sun angle and cloud cover can alter light intensity by orders of magnitude on a second-to-minute scale, and shading by higher leaves can further modify the temporal fluctuations experienced by individual leaves. Several C_4_ species form dense canopies with extensive self-shading where sunflecks can provide up to 90% of the energy for photochemistry ( Tang et al. 1988; Pearcy 1990; Zhu et al. 2004; Way and Pearcy 2012; Slattery et al. 2018. Since photosynthetic responses to changes in light are not instantaneous, fluctuating light has been identified as an area of improvement for crop productivity (Pearcy 1990; Long et al. 2006; Lawson et al. 2012; Kromdijk et al. 2016; Taylor and Long 2017; Kaiser et al. 2018; Acevedo-Siaca et al. 2020). Although recent studies have begun to characterize the C_4_ response (Kubásek et al. 2013, Lee et al. 2022, Li et al. 2021, Pignon et al. 2021), most of our understanding of the limitations of photosynthesis under fluctuating light still comes from C_3_ species (Pearcy 1990; Pearcy et al. 1997; Kaiser et al. 2018). Despite the undeniable global importance of C_4_ crops, with maize (*Zea mays*) and sugarcane (*Saccharum officinarum*) alone accounting for over 30% of global agricultural production (FAO 2020), the impact of the CO_2_ concentrating C_4_ acid shuttle on photosynthetic performance in dynamic light remains understudied (Slattery et al. 2018; Sales et al. 2021).

C_4_ photosynthesis is a remarkably ubiquitous adaptation that has evolved independently at least 66 times in angiosperms (Kellogg 2013) and typically leads to faster photosynthetic rates, higher yields, and greater water use efficiency than the ancestral C_3_ pathway (Kiniry et al. 1989; Sage 2004). Most C_4_ species operate their carbon concentrating mechanism (CCM) by compartmentalizing initial carbon fixation and assimilation between the morphologically distinct mesophyll (M) and bundle sheath (BS) cells, arranged concentrically around the leaf vasculature in “Kranz” anatomy. In the cytosol of M cells, CO_2_ is rapidly converted to bicarbonate by carbonic anhydrase and fixed by phosphoenolpyruvate carboxylase (PEPC) into a 4-carbon oxaloacetate molecule that is further reduced into more stable metabolites malate (Mal) or aspartate (Asp) for transport into the BS. The 4-carbon molecules are decarboxylated in the BS to concentrate CO_2_ around the carbon-fixing enzyme ribulose 1,5-biphosphate carboxylase/oxygenase (Rubisco) and thus enhance photosynthesis by suppressing Rubisco's alternative oxygenation reaction and resulting photorespiration, which consumes energy and reducing equivalents and re-releases CO_2_ (Leegood 2002). Reduced carbon in the form of alanine (Ala) or pyruvate is then transported back to the M cells, where phosphoenolpyruvate (PEP) is regenerated at the cost of ATP, imposing an additional cost on C_4_ metabolism. The specific transport metabolites and enzymes of the C_4_ pathway vary, and whilst species have been traditionally classified based on predominant decarboxylases nicotinamide adenine dinucleotide-malic enzyme (NAD-ME), nicotinamide adenine dinucleotide phosphate-malic enzyme (NADP-ME), and phosphoenolpyruvate carboxykinase (PEPCK) (Hatch et al. 1975), there is a growing consensus that different decarboxylating enzymes often operate in conjunction, with PEPCK likely acting predominantly as a supplementary pathway (Calsa and Figueira 2007; Furbank 2011; Pick et al. 2013; Sales et al. 2018). Crucially, intercellular transport of C_4_ intermediates is driven by diffusion, making the establishment of high metabolic gradients a requirement for the operation of the CCM (Lilley et al. 1977; Leegood and Furbank 1984; Stitt et al. 1985; Arrivault et al. 2017), although model simulations suggest mixed C_4_ pathways could be less reliant on large metabolite pools (Wang et al. 2014a).

Studies conducted on C_3_ species show that photosynthetic response to fluctuating light is restricted by several factors: slow stomatal opening reduces CO_2_ diffusive transfer into the leaf and slow stomatal closing decreases water use efficiency (McAusland et al. 2016), Rubisco activation and the regeneration of ribulose-1,5-biphosphate (RuBP) delay C_3_ cycle activity (Pearcy and Seemann 1990; Sassenrath-Cole and Pearcy 1992; Mott and Woodrow 2000), and the speed of up- and down-regulation of photoprotection lowers light use efficiency (Niu et al. 2022; Zhu et al. 2004). Although said limitations exist irrespective of the photosynthetic pathway, C_4_ species have the additional challenge of coordinating the C_3_ and C_4_ cycles (Kromdijk et al. 2014), and the specifics of the C_4_ response to fluctuating light are not yet fully understood (Kaiser et al. 2018; Slattery et al. 2018). Two apparently contradictory hypotheses can be found in the literature—where C_4_ photosynthesis is suggested to be either less (Kubásek et al. 2013) or more efficient (Stitt and Zhu 2014) under fluctuating light than the ancestral C_3_ form.

The first hypothesis suggests that C_4_ species are more negatively impacted by sudden changes in light intensity due to the C_3_ and C_4_ cycles temporarily operating asynchronously (Sage and McKown 2006). Fluctuations in light could disrupt the buildup of metabolic gradients necessary for the effective operation of the CCM, leading to impaired suppression of photorespiration and reduced photosynthetic efficiency (Kromdijk et al. 2010; Slattery et al. 2018). Alternatively, if the CCM is faster to activate during light induction than the C_3_ cycle there could be transient over-pumping of CO_2_ and an increase in BS leakiness—where CO_2_ diffuses out of BS cells back into M cells, raising the energetic cost of carbon fixation due to the futile cycling of PEP. Greater BS leakiness during induction relative to a steady state has been previously observed in maize and sorghum (*Sorghum bicolor*) (Wang et al. 2022). Lags in CCM deactivation during transitions to lower light could also increase leakiness and reduce quantum yields if Mal/Asp accumulated in the BS is decarboxylated despite insufficient C_3_ cycle activity. In further support for the “negative effect” hypothesis, previous studies have found assimilation rates under fluctuating light relative to steady state to be almost 4 times lower in C_4_ compared to C_3_ species due to slower photosynthetic induction (Li et al. 2021), as well as a more pronounced reduction of biomass in C_4_ than C_3_ plants grown under fluctuating compared to steady light conditions (Kubásek et al. 2013).

The second hypothesis instead posits that C_4_ species are better able to buffer against sudden changes in light intensity because the large metabolite pools required to drive the CCM can store and release ATP and reduce equivalents (Stitt and Zhu 2014). The reversible reactions linking the exchange of 3-phosphoglyceric acid (3-PGA) and triose phosphates (TP) between M and BS cells could provide or consume ATP and NADPH to support the C_3_ cycle (Leegood and von Caemmerer 1989) and mixed C_4_ pathways could transiently enhance Mal over Asp decarboxylation to temporarily increase transport of redox equivalents into the BS (Wang et al. 2014a). In favor of the “positive effect” hypothesis, some of the highest postillumination CO_2_ fixation rates have been found in C_4_ species (Laisk and Edwards 1997), and a recent study on grasses recorded higher rates of carbon assimilation in C_4_ over C_3_ species under fluctuating light due to slower decreases in photosynthetic capacity during high-to-low-light transitions compared to steady state values (Lee et al. 2022).

Although seemingly opposing, there are indications that both hypotheses may coexist. Features of C_4_ biochemistry could have mixed effects—the need to establish large metabolite pools could slow photosynthetic induction but enable higher rates of assimilation upon transitioning to lower light intensity. Slattery et al. (2018) estimated the buffering capacity of C_4_ photosynthesis to be limited to 10 to 15 s based on maize metabolite pool sampling (Arrivault et al. 2017) and suggested that the specific C_4_ response could thus depend on the length of light fluctuations. A time-sensitive response could account for the different responses observed between sunflecks (Laisk and Edwards 1997) and longer fluctuations (Kubásek et al. 2013; Li et al. 2021). However, the different light treatments and species used across studies make it difficult to draw clear conclusions. This is further complicated by C_4_ subtype-specific responses like the postillumination CO_2_ burst observed in NAD-ME species (Krall and Pearcy 1993; Lee et al. 2022), as well as phylogenetic distance, which can strongly confound comparisons between photosynthetic pathways (Taylor et al. 2010), leading to the inappropriate association of species-specific phenomena with the presence or absence of the C_4_ pathway.

In this study, we compared the photosynthetic response to fluctuating light in relation to steady state across 3 phylogenetically linked pairs of C_3_ and C_4_ species from *Alloteropsis* (C_3_*Alloteropsis semialata GMT* & C_4_*Alloteropsis semialata MDG*), *Flaveria* (C_3_*Flaveria cronquistii* & C_4_*Flaveria bidentis*), and *Cleome* (C_3_*Tarenaya hassleriana* & C_4_*Gynandropsis gynandra*) genera representative of monocots and dicots, and of all 3 C_4_ decarboxylase subgroups. Leaves were subjected to a 1-hour fluctuating light treatment consisting of repetitive stepwise changes in light intensity from 800 to 100 *µ*mol m^−2^ s^−1^ photon flux density (PFD), with 3 different times between fluctuations being tested: 6, 30, and 300 s. To evaluate the impact of photorespiration on the responses to fluctuating light, experiments were conducted under both 2% and 21% oxygen concentration. We hypothesized that (i) after a transition to low light, C_4_ species will be better able to sustain photosynthetic rates than C_3_ species, even when photorespiration is suppressed, (ii) C_4_ species will incur a higher penalty during transitions to high light; and (iii) the impact of both effects will be inversely associated with fluctuation length.

## Results

### Steady-state responses of CO_2_ assimilation in paired C_3_ and C_4_ species are consistent with well-established differences between photosynthetic pathways

**Figure 1.**
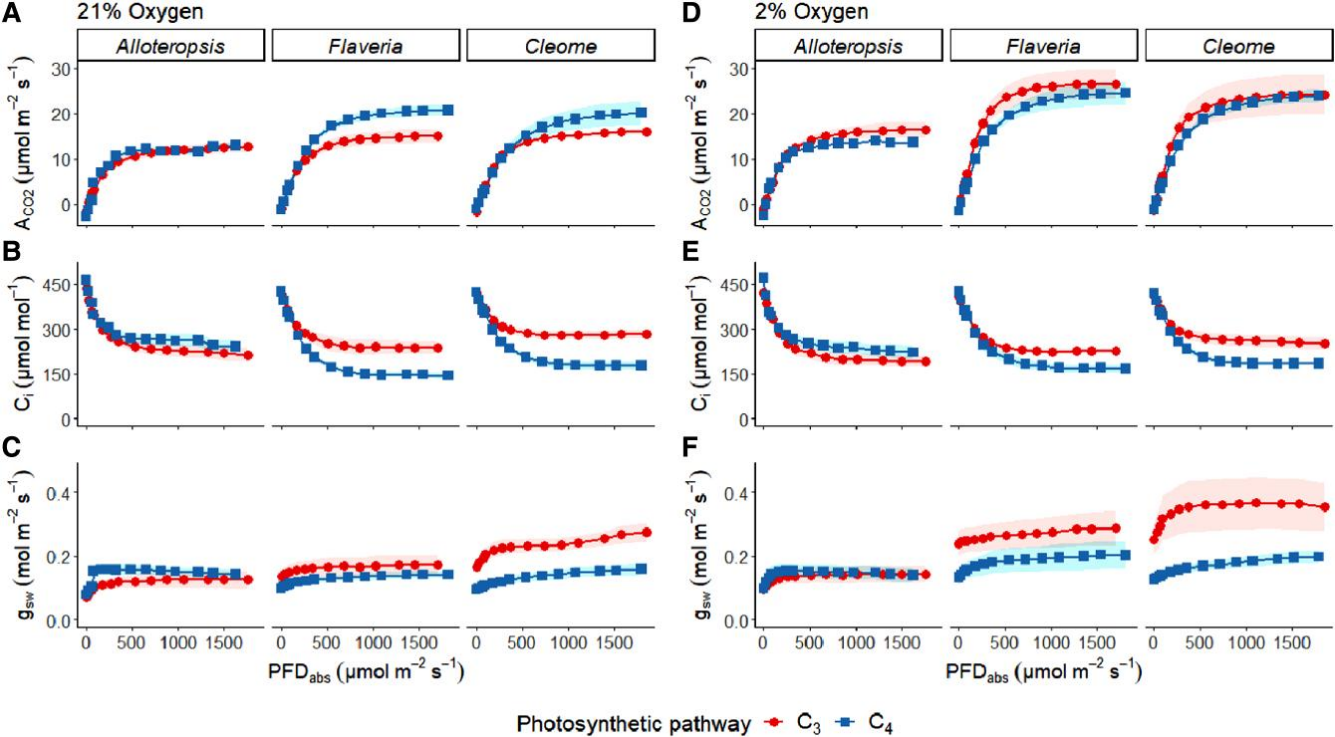
Light response curves of phylogenetically linked C_3_ and C_4_*Alloteropsis*, *Flaveria*, and *Cleome* species at 21% and 2% O_2_. As a function of absorbed photon flux density (PFD_abs_), plots show net CO_2_ assimilation (A_CO2_, **A, D**), intercellular CO_2_ concentration (C_i_, **B, E**), and stomatal conductance to water vapor (g_sw_, **C, F**). Ribbons represent standard error of the mean (*n* = 5).

To provide a baseline for comparing CO_2_ assimilation rates between 3 pairs of closely related C_3_ and C_4_ species under fluctuating light, first, the steady state light response of photosynthetic gas exchange was measured under 21% and 2% O_2_ to minimize photorespiration (Fig. 1). We focus here on photosynthetic parameters at the light intensities that were used in subsequent fluctuating light treatments, 800 and 100 *µ*mol m^−2^ s^−1^ PFD (Table 1). Two-way ANOVA was used to assess the effects of photosynthetic pathway, oxygen concentration, and their interactions within each genus (Table 2). At both light intensities C_3_ species trended toward higher assimilation values and quantum yields under photorespiration-suppressing conditions than C_4_ species.

**Table 1.** Photosynthetic parameters estimated from steady-state light response curves under 21% and 2% O_2_ in phylogenetically linked C_3_ and C_4_*Alloteropsis*, *Flaveria*, and *Cleome* species. Respiration in the light (*Rd*) was calculated by fitting the light response curves with a nonrectangular hyperbola. Values for *800* A_CO2_, *100* A_CO2_, C_i 800_, C_i 100_, *800* ΦCO_2_, and *100* ΦCO_2_ were taken at PFD = 800 *µ*mol m^−2^ s^−1^ and PFD = 100 *µ*mol m^−2^ s^−1^. The means and standard error of the mean are shown (*n* = 5)

		21% oxygen		2% oxygen	
Genus	Parameter	C_3_	C_4_	C_3_	C_4_
*Alloteropsis*	*800* A_CO2_ (µmol m^−2^ s^−1^)	11.3 ± 0.9	12.3 ± 0.5	15.0 ± 1.2	13.1 ± 0.1
	*100* A_CO2_ (µmol m^−2^ s^−1^)	3.2 ± 0.5	4.7 ± 0.5	4.8 ± 0.7	4.7 ± 0.7
	C_i 800_ (µmol mol^−1^)	235 ± 20	265 ± 15	207 ± 21	245 ± 20
	C_i 100_ (µmol mol^−1^)	348 ± 9	349 ± 2	334 ± 9	345 ± 2
	*800* ΦCO_2_	0.017 ± 0.003	0.021 ± 0.002	0.025 ± 0.006	0.022 ± 0.00
	*100* ΦCO_2_	0.047 ± 0.012	0.075 ± 0.014	0.065 ± 0.005	0.076 ± 0.018
	*Rd*	0.6 ± 0.1	1.7 ± 0.3	1.5 ± 0.3	1.5 ± 0.1
*Flaveria*	*800* A_CO2_ (µmol m^−2^ s^−1^)	13.8 ± 1.4	18.7 ± 1.0	25.0 ± 2.9	21.5 ± 1.7
	*100* A_CO2_ (µmol m^−2^ s^−1^)	3.9 ± 0.2	4.2 ± 0.3	6.6 ± 0.3	4.7 ± 0.4
	C_i 800_ (µmol mol^−1^)	246 ± 25	157 ± 8	230 ± 8	182 ± 18
	C_i 100_ (µmol mol^−1^)	352 ± 6	339 ± 4	351 ± 5	343 ± 9
	*800* ΦCO_2_	0.022 ± 0.004	0.027 ± 0.00	0.038 ± 0.01	0.030 ± 0.005
	*100* ΦCO_2_	0.057 ± 0.005	0.057 ± 0.007	0.085 ± 0.01	0.056 ± 0.009
	*Rd*	0.4 ± 0.4	0.8 ± 0.3	0.6 ± 0.2	0.4 ± 0.2
*Cleome*	*800* A_CO2_ (µmol m^−2^ s^−1^)	14.6 ± 0.5	23.5 ± 2.0	27.3 ± 4.0	27.4 ± 0.9
	*100* A_CO2_ (µmol m^−2^ s^−1^)	4.1 ± 0.2	3.2 ± 0.4	6.2 ± 0.6	4.7 ± 0.5
	C_i 800_ (µmol mol^−1^)	281 ± 14	190 ± 12	266 ± 21	192 ± 9
	C_i 100_ (µmol mol^−1^)	363 ± 7	352 ± 3	358 ± 18	344 ± 7
	*800* ΦCO_2_	0.020 ± 0.001	0.026 ± 0.006	0.033 ± 0.012	0.030 ± 0.003
	*100* ΦCO_2_	0.048 ± 0.004	0.056 ± 0.011	0.090 ± 0.016	0.061 ± 0.012
	*Rd*	1.5 ± 1.0	1.2 ± 0.4	2.1 ± 0.7	0.8 ± 0.5

**Table 2.** ANOVA table of light response curve parameters at 100 and 800 *µ*mol m^−2^ s^−1^ PFD for phylogenetically linked C_3_ and C_4_*Alloteropsis*, *Flaveria*, and *Cleome* species. Photosynthetic pathway, PP. O_2_ concentration, [O_2_]. Interaction effect, PP:[O_2_]. The table shows degrees of freedom; *F*-value; and *P*-value. Significant *P*-values (*a* < 0.05) are shown in bold

	*Alloteropsis*			*Flaveria*			*Cleome*		
Parameter	PP	[O_2_]	PP:[O_2_]	PP	[O_2_]	PP:[O_2_]	PP	[O_2_]	PP:[O_2_]
*800* A_CO2_	1.16; 0.35; 0.56	1.16; 7.77; **0.01**	1.16; 3.24; 0.09	1.16; 0.12; 0.73	1.16; 13.48; **0.01**	1.16; 4.83; **0.04**	1.15; 0.01; 0.91	1.15; 6.18; **0.02**	1.15; 0.82; 0.28
*100* A_CO2_	1.16; 1.21; 0.29	1.16; 1.60; 0.22	1.16; 1.56; 0.23	1.16; 7.01; **0.02**	1.16; 25.64; **≤ 0.001**	1.16; 11.38; **0.01**	1.15; 7.28; **0.02**	1.15; 15.06; **0.01**	1.15; 0.43; 0.52
C_i 800_	1.16; 3.21; 0.09	1.16; 1.62; 0.22	1.16; 0.05; 0.83	1.16; 17.63; **≤ 0.001**	1.16; 0.09; 0.76	1.16; 1.63; 0.22	1.16; 31.44; **≤ 0.001**	1.16; 0.19; 0.66	1.16; 1.33; 0.58
C_i 100_	1.16; 0.87; 0.36	1.16; 1.96; 0.40	1.16; 0.74; 0.40	1.16; 2.52; 0.13	1.16; 0.11; 0.75	1.16; 0.16; 0.70	1.16; 3.65; 0.07	1.16; 1.01; 0.33	1.16; 0.04; 0.84
*800* ΦCO_2_	1.16; 0.22; 0.07	1.16; 8.12; **0.01**	1.16; 4.47; **0.05**	1.16; 0.13; 0.72	1.16; 11.90; **0.01**	1.16; 5.95; **0.03**	1.16; 0.19; 0.67	1.16; 6.96; **0.02**	1.16; 2.18; 0.15
*100* ΦCO_2_	1.16; 11.03; **0.01**	1.16; 2.67; 0.12	1.16; 2.04; 0.17	1.16; 15.56; **0.01**	1.16; 13.93; **0.01**	1.16; 16.53; **≤ 0.001**	1.16; 4.50; **0.05**	1.16; 21.01; **≤ 0.001**	1.16; 12.72; **0.01**

At 800 *µ*mol m^−2^ s^−1^ PFD, net CO_2_ assimilation (A_CO2_) values in C_4_*A. semialata MDG* (12.3 ± 0.5 *µ*mol m^−2^ s^−1^), C_4_*F. bidentis* (18.7 ± 1.0 *µ*mol m^−2^ s^−1^), and C_4_*G. gynandra* (23.5 ± 2.0 *µ*mol m^−2^ s^−1^) were, respectively, higher than phylogenetic pairs C_3_*A. semialata GMT* (11.3 ± 0.9 *µ*mol m^−2^ s^−1^), C_3_*F. cronquistii* (13.8 ± 1.4 *µ*mol m^−2^ s^−1^), and C_3_*T. hassleriana* (14.6 ± 0.5 *µ*mol m^−2^ s^−1^). Under 2% oxygen, photosynthetic rates increased by 33% in C_3_*A. semialata GMT*, 81% in C_3_*F. cronquistii,* and 86% in C_3_*T. hassleriana*, compared to a more modest increase of 6%, 15%, and 16% in their respective C_4_ pairs. Nevertheless, the effect of O_2_ at 800 *µ*mol m^−2^ s^−1^ PFD on A_CO2_ as well as on quantum yield of CO_2_ assimilation (ΦCO_2_) was significant across all 3 genera (*P* ≤ 0.02, Table 2).

At 100 *µ*mol m^−2^ s^−1^ PFD, the effects of photosynthetic pathway, O_2_ concentration, and their interaction on A_CO2_ varied between genera. In *Alloteropsis,* none of the effects were significant, although C_4_*A. semialata MDG* (4.7 ± 0.5 *µ*mol m^−2^ s^−1^) showed slightly higher A_CO2_ values than C_3_*A. semialata GMT* (3.2 ± 0.5 *µ*mol m^−2^ s^−1^) under ambient O_2_. In *Flaveria,* values of A_CO2_ were similar under ambient O_2_ in C_4_*F. bidentis* (4.2 ± 0.3 *µ*mol m^−2^ s^−1^), and C_3_*F. cronquistii* (3.9 ± 0.2 *µ*mol m^−2^ s^−1^), but significantly higher in C_3_*F. cronquistii* under 2% O_2_ (4.7 ± 0.4 vs 6.6 ± 0.3 *μ*mol m^−2^ s^−1^, respectively, Table 2). As a result, both the main effects of O_2_ and the photosynthetic pathway as well as their interaction on both A_CO2_ and ΦCO_2_ were significant in *Flaveria* (*P* ≤ 0.02). In *Cleome*, A_CO2_ under 21% O_2_ was lower in C_4_*G. gynandra* (3.2 ± 0.4 *µ*mol m^−2^ s^−1^) than in C_3_*T. hassleriana* (4.1 ± 0.2 *µ*mol m^−2^ s^−1^) and both rates increased under 2% O_2_ (4.7 ± 0.5 and 6.2 ± 0.6 *µ*mol m^−2^ s^−1^, respectively), resulting in significant effects of both photosynthetic pathway and O_2_ concentration (*P* ≤ 0.02, Table 2).

### CO_2_ assimilation under fluctuating light differs between genera and is significantly affected by fluctuation frequency, photosynthetic pathway, and oxygen concentration

**Figure 2.**
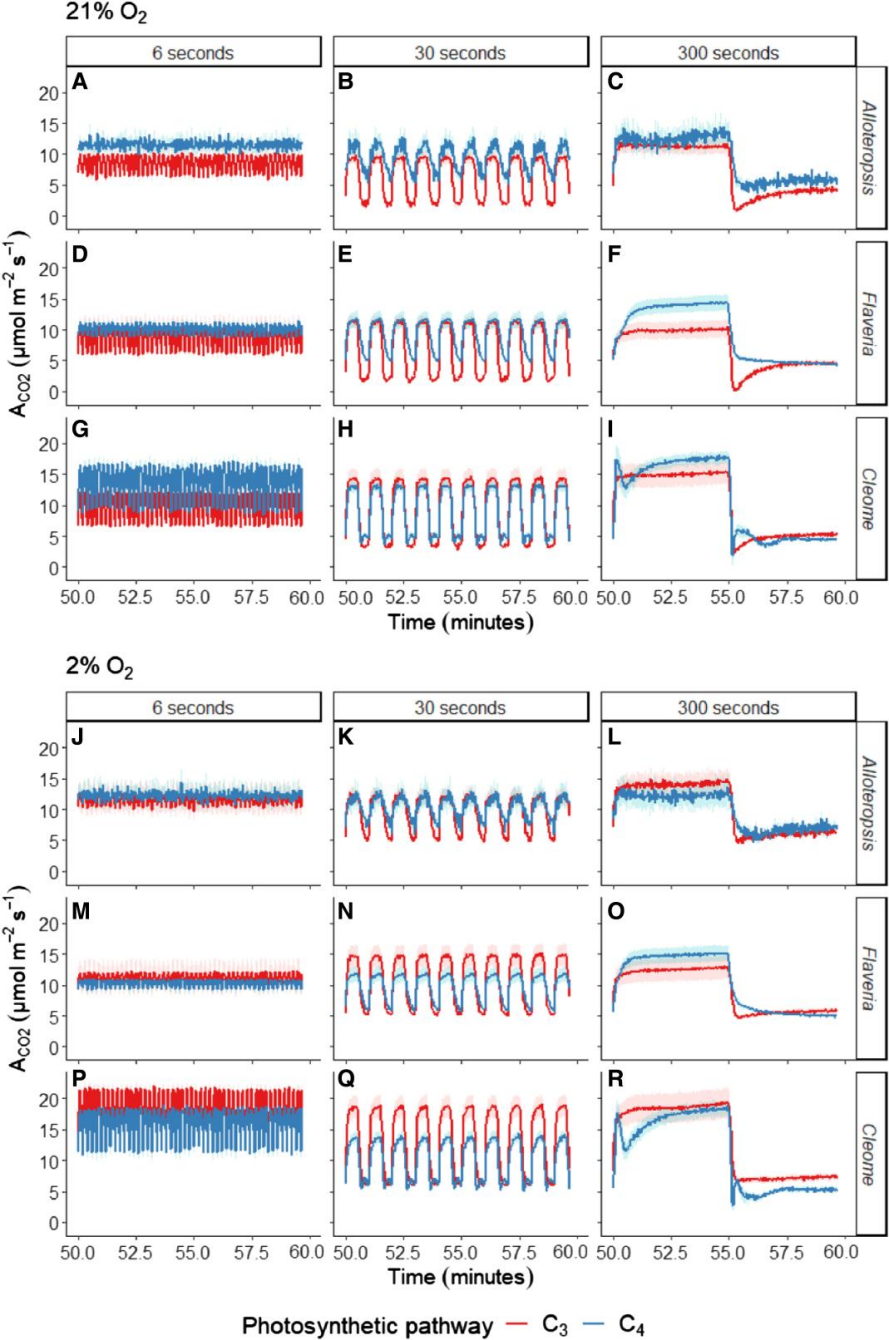
Net CO_2_ assimilation (A_CO2_) in phylogenetically linked C_3_ and C_4_*Alloteropsis*, *Flaveria*, and *Cleome* species under 3 different fluctuating light regimes at 21% (**A-I**) and 2% O_2_ (**J-R**). Each light regime consisted of alternating 800 and 100 *µ*mol m^−2^ s^−1^ PFD periods, where each light step lasted 6, 30, or 300 s before changing. Treatments were started after leaves were acclimated at 150 *µ*mol m^−2^ s^−1^ PFD and lasted 1 h, data was analyzed from minutes 50 to 60 of each treatment. Ribbons represent the standard error of the mean (*n* = 5). The full time series is shown in Supplemental Fig. S1.

After characterizing steady-state light responses, CO_2_ assimilation rates were measured in response to 3 different 1-h fluctuating light treatments. Each treatment consisted of acclimation at 150 *µ*mol m^−2^ s^−1^ PFD, followed by repetitive stepwise changes between 800 and 100 *µ*mol m^−2^ s^−1^ PFD where each light step lasted 6, 30, or 300 s. Data from minutes 50 to 60 of each treatment (Fig. 2) were used for analysis to exclude the effect of initial induction (for the complete time series see Supplemental Fig. S1). Since dynamic measurements violate the steady state assumption underlying default rate equations, a dynamic correction was applied using principles of mass balance recently outlined by Saathoff and Welles (2021). The fluctuating light response generally consisted of a rise in A_CO2_ toward a steady state in the 800 *µ*mol m^−2^ s^−1^ PFD period and a subsequent decrease during the 100 *µ*mol m^−2^ s^−1^ PFD period. A_CO2_ was strongly increased under 2% O_2_ in the C_3_ species, but much less so in the C_4_ species. More subtle patterns varied by photosynthetic type, fluctuation length, and between genera as described below.

After transitioning from 800 to 100 *µ*mol m^−2^ s^−1^ PFD, photosynthetic assimilation in C_4_*A. semialata MDG* and C_4_*F. bidentis* decreased more gradually compared to the immediate drop followed by a rise toward steady state observed in C_3_*A. semialata GMT,* C_3_*F. cronquistii* and C_3_*T. hassleriana*. This drop in assimilation in the C_3_ species, known as the postillumination CO_2_ burst (PIB), has previously been associated with photorespiration (Forrester et al. 1966, Wynn et al. 1973) and indeed was suppressed in all C_3_ species under 2% oxygen (most easily seen in Fig. 2 C/F/I compared to Fig. 2 L/O/R). The slower decrease of A_CO2_ in C_4_*A. semialata MDG* and C_4_*F. bidentis* was evident under 30 s (Fig. 2 B/E) and 300 s light steps (Fig. 2 C/F) in both oxygen concentrations, whilst under 6 s light steps (Fig. 2 A/D) A_CO2_ at 100 *µ*mol m^−2^ s^−1^ stayed closer to rates obtained during the 800 *µ*mol m^−2^ s^−1^ PFD periods, suggesting a less substantial initial decline. Irrespective of oxygen concentration and unlike the delayed decrease observed in the other C_4_ species, in C­_4_*G. gynandra* an initial dip in A_CO2_ was observed immediately following the transition to low light (Fig. 2 I/R).

Following the transition from 100 to 800 *µ*mol m^−2^ s^−1^ PFD, induction patterns strongly varied between the 3 C_4_ species, in contrast with the more consistent patterns observed in C_3_ species. C_4_*F. bidentis* and C_4_*G. gynandra* had higher A_CO2_ during the 300 s light steps than C_3_*F. bidentis* and C_3_*T. hassleriana* (Fig. 2 C/F), but similar or lower A_CO2_ than their C_3_ counterparts during the 30 s light step (Fig. 2 B/E) even under 21% oxygen, suggesting a comparatively greater lag in photosynthetic induction. However, the induction of A_CO2_ in C_4_*A. semialata MDG* was very similar to that of C_3_*A. semialata GMT*. In C_4_*G. gynandra* a strong temporary depression in A_CO2_ was observed after an initial sharp increase upon exposure to higher light under the 300 s light step that was not affected by the suppression of photorespiration by low oxygen (see Fig. 2 I vs R).

**Figure 3.**
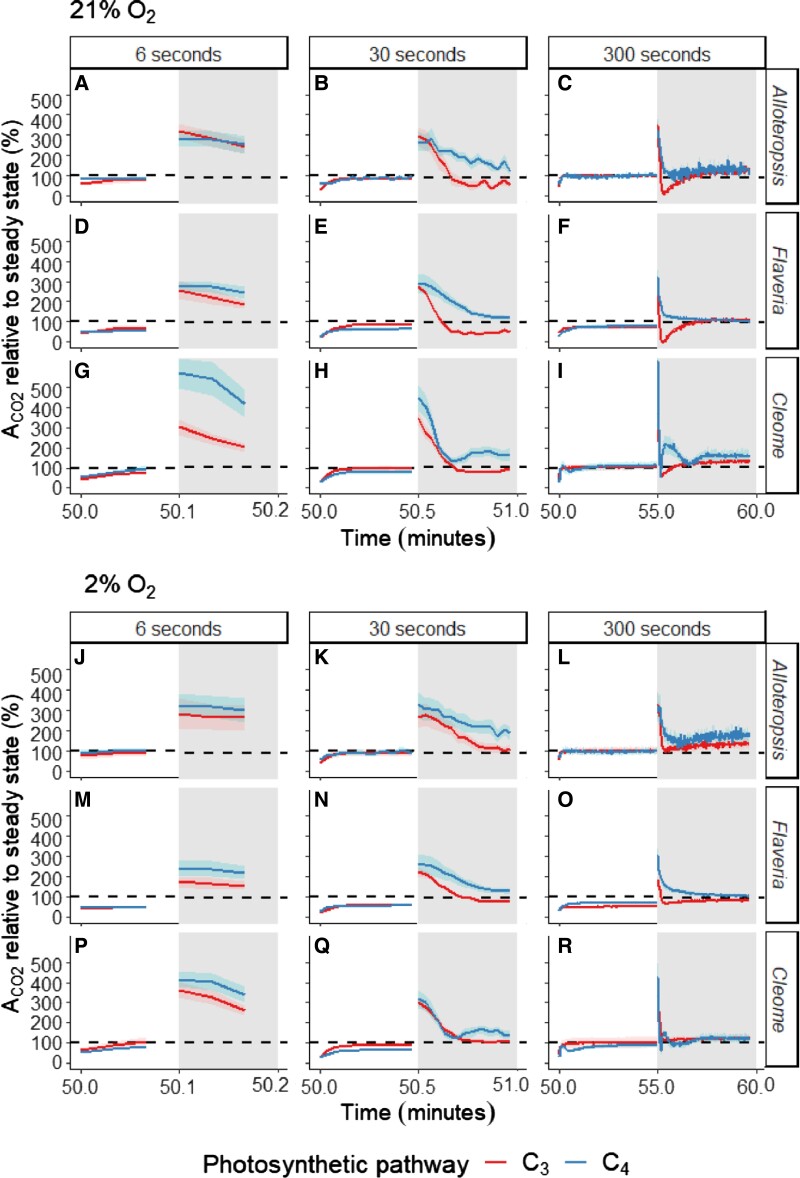
Net CO_2_ assimilation (A_CO2_) relative to steady state (%) across the 800 and 100 *µ*mol m^−2^ s^−1^ PFD light steps of differing lengths starting at the 50 min mark, in white and shaded, respectively. Depending on the fluctuating light treatment, subplots are showing one complete fluctuation of 12, 60, or 600 s. Values represent A_CO2_ at a given point in the fluctuating light treatment relative to A_CO2_ obtained from steady-state light response curves at the light intensity of each period in phylogenetically linked C_3_ and C_4_*Alloteropsis*, *Flaveria*, and *Cleome* species at 21% (**A-I**) and 2% O_2_ (**J-R**). The dashed line represents 100%, where assimilation would be exactly that of steady state. Ribbons represent the standard error of the mean (*n* = 5). The corresponding absolute assimilation values are provided in Supplemental Fig. S2.

To compare photosynthetic performance between C_3_ and C_4_ species whilst accounting for their different steady-state photosynthetic capacities, A_CO2_ was expressed as a relative percentage of steady-state values obtained from light response curves (shown in Fig. 3). The corresponding absolute carbon assimilation values are provided in Supplemental Fig. S2, and steady state values in Fig. 1). This analysis showed clear, systematic differences between C_3_ and C_4_ species during the 100 *µ*mol m^−2^ s^−1^ PFD steps, where all C_4_ species were able to sustain higher A_CO2_ under 100 *µ*mol m^−2^ s^−1^ relative to steady state than their matching C_3_ counterparts under both 21% and 2% oxygen. However, no systematic difference between photosynthetic types was apparent during the 800 *µ*mol m^−2^ s^−1^ PFD steps (Supplemental Fig. S3).

### Stimulation of CO_2_ assimilation at low light is most prominent in short light steps and significantly greater in C_4_ compared to C_3_ species

**Figure 4.**
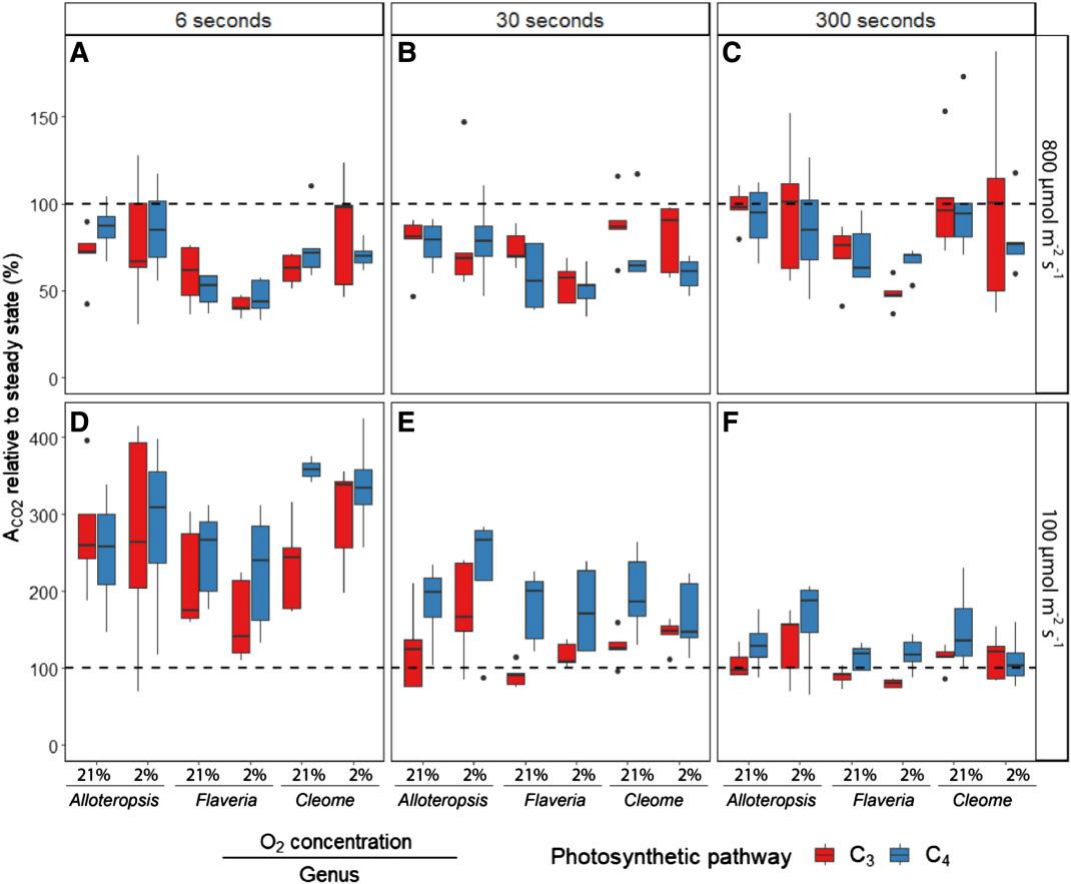
Boxplots of net CO_2_ assimilation (A_CO2_) relative to steady state (%) under the 800 (**A–C**) and 100 *µ*mol m^−2^ s^−1^ PFD (**D–F**) light steps of the fluctuating light regimes. Each regime consisted of alternating 800 and 100 *µ*mol m^−2^ s^−1^ PFD periods, where each light step lasted 6, 30, or 300 s. For each period, A_CO2_ across the time series for phylogenetically linked C_3_ and C_4_*Alloteropsis*, *Flaveria*, and *Cleome* species at 21% or 2% O_2_ was calculated as a percentage of steady state values obtained from light response curves at the same light intensity and O_2_ concentration. The dashed line represents 100%, where the assimilation rate would equal steady state. Box edges represent the lower and upper quartiles, the solid line indicates the median, and points represent outliers beyond 1.5 times the interquartile range (*n* = 5 for each combination of species/oxygen). Three-way ANOVA (Table 4) was used to test the effect of photosynthetic pathway, fluctuating length, O_2_ concentration, and their interaction on A_CO2_ relative to steady state in *Alloteropsis, Flaveria*, and *Cleome*. The corresponding absolute assimilation values are shown in Supplemental Fig. S4.

To quantify the stimulation of A_CO2_ during the 100 *µ*mol m^−2^ s^−1^ PFD steps of the fluctuations, the average A_CO2_ during the 100 *µ*mol m^−2^ s^−1^ PFD steps was normalized against the steady state rate at the same intensity (Fig. 4 D-F, for a boxplot of the absolute A_CO2_ values, see Supplemental Fig. S4 E-G). This analysis showed that assimilation was higher than steady-state (as seen in Fig. 3 gray half, values greater than 100%) in all species immediately following the transition but declined with duration of the light steps (Table 3, *P* ≤ 0.001 for all). In addition, the relative stimulation compared to steady-state values was consistently significantly greater in the C_4_ species compared to the C_3_ species in the *Flaveria* and *Cleome* pairs (*Flaveria P* ≤ 0.01; *Cleome P* ≤ 0.001, Table 3). Although not significant, a similar trend was observed for the *Alloteropsis* pair at 300 and 30 s, but not at 6 s light steps.

**Table 3.** ANOVA table of percentage A_CO2_ relative to steady state during the 2 different light steps of the light fluctuation treatments for phylogenetically linked C_3_ and C_4_*Alloteropsis*, *Flaveria*, and *Cleome* species. Photosynthetic pathway, PP. Fluctuating length, fl. O_2_ concentration, [O_2_]. Interaction effects, PP:fl, PP:[O_2_], fl:[O_2_], and PP:fl:[O_2_]. The table shows degrees of freedom; *F*-value; and *P*-value. Significant *P*-values (*a* < 0.05) are shown in bold

Light period (µmol m^−2^ s^−1^)	Genus	PP	fl	[O_2_]	PP:fl	PP:[O_2_]	fl:[O_2_]	PP:fl:[O_2_]
800	*Alloteropsis*	1.52; 0.01; 0.92	1.52; 3.64; 0.06	1.52; 0.01; 0.93	1.52; 0.96; 0.33	1.52; 0.13; 0.72	1.52; 0.19; 0.66	1.52; 0.00; 0.98
	*Flaveria*	1.52; 0.07; 0.80	1.52; 8.58; **0.01**	1.52; 13.90; **≤ 0.001**	1.52; 4.23; **0.04**	1.52; 4.65; **0.04**	1.52; 0.04; 0.84	1.52; 0.09; 0.76
	*Cleome*	1.52; 1.32; 0.25	1.52; 7.96; **0.01**	1.52; 0.53; 0.47	1.52; 0.00; 0.98	1.52; 1.54; 0.22	1.52; 0.72; 0.40	1.52; 0.00; 0.95
100	*Alloteropsis*	1.52; 1.15; 0.29	1.52; 15.30; **≤ 0.001**	1.52; 1.48; 0.23	1.52; 0.01; 0.90	1.52; 0.07; 0.79	1.52; 0.00; 0.96	1.52; 0.02; 0.89
	*Flaveria*	1.52; 14.20; **≤ 0.001**	1.52; 31.18; **≤ 0.001**	1.52; 0.50; 0.48	1.52; 0.94; 0.33	1.52; 0.03; 0.87	1.52; 0.24; 0.62	1.52; 0.02; 0.89
	*Cleome*	1.52; 7.65; **0.01**	1.52; 25.08; **≤ 0.001**	1.52; 0.50; 0.48	1.52; 2.64; 0.11	1.52; 3.06; 0.08	1.52; 0.00; 0.99	1.52; 0.53; 0.46

Three-way ANOVA (Table 3) showed A_CO2_ relative to steady state was significantly affected by fluctuation length in all genera (*P* ≤ 0.001 for all, Table 3), as well as by photosynthetic pathway in *Flaveria* and *Cleome* (*Flaveria P* ≤ 0.01; *Cleome P* ≤ 0.001, Table 3). Overall, although all species had greater CO_2_ fixation during the 100 *µ*mol m^−2^ s^−1^ PFD periods than under steady state, the effect was time sensitive and therefore more significant during shorter light steps. In addition, C_4_ species were able to sustain the higher rates for longer than their C_3_ counterparts. The greatest increases in relative assimilation occurred during the 6 s light steps (Fig. 4 D) where C_4_ species were on average 329% of steady state A_CO2_ compared to 242% in C_3_ species under 21% oxygen and similarly 290% in C_4_ vs 243% in C_3_ at 2% oxygen. Under 30 s light steps (Fig. 4 E) the stimulation of A_CO2_ relative to steady state was less pronounced than 6 s, but still substantially higher in C_4_ species at 187% compared to C_3_ species at 114% of steady state A_CO2_ at 21% O_2_, and 190% vs 146% of steady state A_CO2_ at 2% O_2_, respectively. The impact of C_4_ photosynthesis was most apparent under these 2 light steps, whereas during the 300 s light steps (Fig. 4 F) the stimulation of A_CO2_ was less evident. Averaged across the 300 s, C_4_ species were operating at 132% relative to steady state A_CO2_ compared to C_3_ species at 103% under ambient oxygen, and at 130% and 109% of steady state A_CO2_ under 2% O_2_, respectively. Interestingly, A_CO2_ relative to steady state was typically higher in C_4_ compared to C_3_ species under both 21% and 2% oxygen and no significant effect of the interaction between photosynthetic pathway and oxygen concentration was found in any of the genera, suggesting a systematic advantage to C_4_ photosynthesis to bridge low-light periods which was still apparent when photorespiration was suppressed.

Despite the fact that photorespiration did not account for the difference in A_CO2_ between C_3_ and C_4_ species during the initial transition to lower light, photorespiratory lagging led to a clear PIB in the C_3_ species, which further exacerbated the decline in A_CO2_ immediately following high light in the measurements under 21% O_2_. To estimate the impact of the PIB on A_CO2_ in the C_3_ species, periods of the 100 *µ*mol m^−2^ s^−1^ PFD light steps where a PIB was evident were compared between 21% and 2% O_2_ (10 to 30 s in the 30 s light steps and 10 to 70 s in the 300 s light steps). Under 21% O_2_ the average A_CO2_ relative to steady state of C_3_ species during those periods was 69% and 61% for the 30 and 300 s light steps, compared to 111% and 112% respectively under 2% O_2_. In contrast, under both oxygen concentrations, the relative assimilation of C_4_ species was consistently greater than 100% across both fluctuation lengths, averaging 150% and 158% under 21% O_2_, and 161% and 151% under 2% over the same 30 and 300 s periods.

**Figure 5.**
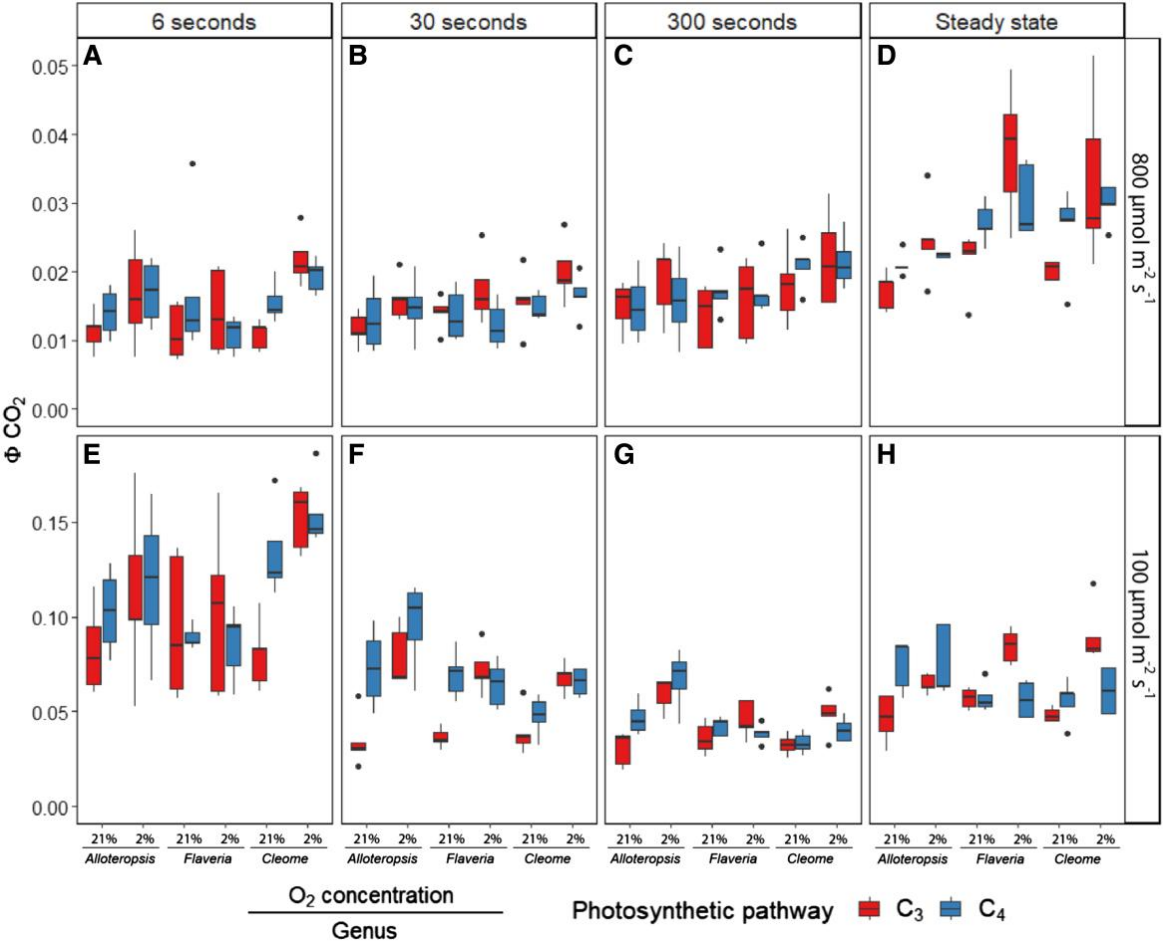
Boxplots of the quantum yield of carbon assimilation (ΦCO_2_) under the 800 (**A–C**) and 100 (**E–H**) µmol m^−2^ s^−1^ PFD periods of the fluctuating light regimes or from steady-state measurements (**D, H**). Each fluctuating regime consisted of alternating 800 and 100 *µ*mol m^−2^ s^−1^ PFD periods, where each light step lasted 6, 30, or 300 s. Box edges represent the lower and upper quartiles, the solid line indicates the median, and points represent outliers beyond 1.5 times the interquartile range (*n* = 5 for each combination of species/measurement condition). Three-way ANOVA was used to test the effect of photosynthetic pathway, fluctuating length, O_2_ concentration, and their interaction on ΦCO_2_ in *Alloteropsis, Flaveria*, and *Cleome* (results shown in Table 5).

To estimate to what extent the low-light stimulation of CO_2_ assimilation was decoupled from photochemical provision of ATP and NADPH, ΦCO_2_ was calculated for each light step (Fig. 5, Table 4). Based on steady-state stoichiometry of electron flow and proton requirements for ATP synthesis and NADPH:ATP energy demands, the theoretical maximum ФCO_2_ has been estimated as 0.111 CO_2_/photon for C_3_ species (Ehleringer and Pearcy 1983), as 0.064 CO_2_/photon for C_4_ NADP-ME and NAD-ME species accounting for estimated BS leakiness (Yin and Struik 2018), and due to the theorized lower energy requirements of mixed C_4_ pathways as 0.075 CO_2_/photon for mixed subtype NADP-ME-PEPCK (Ishikawa et al. 2016, Yin and Struik 2021). Here we consider observations of quantum yields exceeding these theoretical maxima as conservative evidence for decoupling. At 6 s light steps, C_3_*F. cronquistii* and C_3_*A. semialata GMT* stayed well below the theoretical maximum, but the ФCO_2_ values of C_3_*T. hassleriana* were significantly higher (Table 4). ФCO_2_ values of C_4_*A. semialata MDG,* C_4_*F. bidentis, and* C_4_*G. gynandra* during the lower light periods of the 6 and 30 s fluctuations were also significantly higher than the theoretical limit, suggesting that the provision of ATP and reductant was not directly coupled to production from the thylakoid reactions. By comparing the ФCO_2_ values with these theoretical maxima, it is possible to estimate the degree of decoupling in units of fixed CO_2_/photon. At 6 s light steps, the theoretical limit was exceeded by 0.043 ± 0.019 CO_2_/photon in C_4_*A. semialata MDG*, 0.022 ± 0.008 CO_2_/photon in C_4_*F. bidentis,* 0.091 ± 0.008 CO_2_/photon in C_4_*G. gynandra* and 0.042 ± 0.008 CO_2_/photon in C_3_*T. hassleriana*. At 30 s light steps, these numbers decreased to 0.021 ± 0.011 CO_2_/photon in C_4_*A. semialata MDG*; 0.008 ± 0.005 CO_2_/photon in C_4_*F. bidentis*; and 0.005 ± 0.003 CO_2_/photon in C_4_*G. gynandra*.

**Table 4.** ANOVA table of ΦCO_2_ during the 2 different light steps of the light fluctuation treatments for phylogenetically linked C_3_ and C_4_*Alloteropsis*, *Flaveria*, and *Cleome* species. Photosynthetic pathway, PP. Fluctuating length, fl. O_2_ concentration, [O_2_]. Interaction effects, PP:fl, PP:[O_2_], fl:[O_2_], and PP:fl:[O_2_]. The table shows degrees of freedom; *F*-value; and *P*-value. Significant *P*-values (*a* < 0.05) are shown in bold

Light period (µmol m^−2^ s^−1^)	Genus	PP	fl	[O_2_]	PP:fl	PP:[O_2_]	fl:[O_2_]	PP:fl:[O_2_]
800	*Alloteropsis*	1.52; 0.00; 0.97	1.52; 2.00; 0.16	1.52; 7.08; **0.01**	1.52; 0.76; 0.39	1.52; 1.22; 0.27	1.52; 0.20; 0.66	1.52; 0.00; 0.96
	*Flaveria*	1.52; 0.10; 0.75	1.52; 2.67; 0.11	1.52; 0.02; 0.89	1.52; 1.17; 0.28	1.52; 4.56; **0.03**	1.52; 0.40; 0.53	1.52; 0.77; 0.38
	*Cleome*	1.52; 0.01; 0.92	1.52; 11.15; **0.01**	1.52; 16.33; **≤ 0.001**	1.52; 0.53; 0.47	1.52; 4.45; **0.04**	1.52; 2.48; 0.12	1.52; 0.17; 0.68
100	*Alloteropsis*	1.52; 5.70; **0.02**	1.52; 23.37; **≤ 0.001**	1.52; 14.02; **≤ 0.001**	1.52; 0.25; 0.62	1.52; 0.94; 0.34	1.52; 0.04; 0.85	1.52; 0.07; 0.79
	*Flaveria*	1.52; 0.01; 0.94	1.52; 34.60; **≤ 0.001**	1.52; 1.28; 0.26	1.52; 0.00; 0.99	1.52; 3.26; 0.08	1.52; 1.15; 0.70	1.52; 0.17; 0.68
	*Cleome*	1.52; 1.04; 0.31	1.52; 38.70; **≤ 0.001**	1.52; 9.40; **0.02**	1.52; 1.21; **0.04**	1.52; 1.81; 0.28	1.52; 1.75; 0.19	1.52; 0.42; 0.52

### Depression of CO_2_ assimilation at high light is not significantly affected by photosynthetic pathway

Unlike in the 100 *µ*mol m^−2^ s^−1^ PFD periods there was no clear trend between the C_3_ and C_4_ response at 800 *µ*mol m^−2^ s^−1^ PFD periods (Fig. 4 A–C, for a boxplot of the corresponding A_CO2_ values, see Supplemental Fig. S4 A–C).

A_CO2_ values during the 800 *µ*mol m^−2^ s^−1^ PFD light steps were lower than under steady state (below 100% line in Fig. 3 and Supplemental Fig. S3). Three-way ANOVA (Table 3) was used to analyze the effects of light step duration, photosynthetic pathway, oxygen concentration, and their interactions on relative A_CO2_. None of these were significant for the *Alloteropsis* subspecies. In *Flaveria*, weakly significant interactions between light step duration and photosynthetic pathway (*P* = 0.04, Table 3), as well as between oxygen concentration and photosynthetic pathway (*P* = 0.04, Table 3) were observed, indicating a more complex conditional impact of photosynthetic pathway on assimilation rate relative to steady state. In C_4_*F. bidentis* relative A_CO2_ gradually increased with light step duration, whereas in C_3_*F. cronquistii* this increase was only observed between 6 and 30 s but not between 30 and 300 s. Whilst relative A_CO2_ was depressed by 2% oxygen in both *Flaveria* species, the effect was more pronounced in C_3_*F. cronquistii*. In *Cleome*, light step duration significantly impacted relative A_CO2_ (*P* = 0.01, Table 3) which increased with duration in both C_3_ and C_4_ species.

Quantum yields during the 800 *µ*mol m^−2^ s^−1^ PFD periods across all fluctuation regimes and oxygen concentrations were lower than steady state across all species (Fig. 5 A–D), indicating reduced efficiency of carbon assimilation. In *Alloteropsis*, oxygen concentration significantly impacted ФCO_2_ (*P* = 0.01, Table 4), with 2% oxygen being associated with higher values. In *Flaveria* and *Cleome* 2% oxygen was associated with higher quantum yields only in the C_3_ species, and lower or similar values in their C_4_ counterparts (significant interactions between O_2_ and photosynthetic pathway *P* ≤ 0.05, Table 4).

## Discussion

### C_4_ species are better able to sustain photosynthetic rates than C_3_ species after a transition to lower light

The effect of fluctuating light on C_4_ relative to C_3_ photosynthesis was systematically evaluated in 3 phylogenetically controlled comparisons using repetitive low- and high-light steps with 3 contrasting durations. The results support the hypothesis that C_4_ species are better able to sustain photosynthetic rates than C_3_ species during the lower light periods of fluctuating light. The theoretical basis for this hypothesis suggests that the large metabolite pools necessary for diffusional transfer between M and BS in C_4_ photosynthesis and the reversible reactions linking these metabolic intermediates can work as a capacitor, providing greater flexibility to respond to variations in light intensity (Leegood and von Caemmerer 1989; Stitt and Zhu 2014). In this study, although all tested species had generally higher carbon assimilation during the 100 *µ*mol m^−2^ s^−1^ PFD periods relative to steady state, C_4_*A. semialata MDG,* C_4_*F. bidentis*, and C_4_*G. gynandra* had higher relative rates than C_3_*A. semialata GMT*, C_3_*F. bidentis,* and C_3_*T. hassleriana* under the same fluctuating light regime (Fig. 4 D-F). As C_4_ species had higher relative assimilation under both 21% and 2% oxygen, the greater stimulation under low light cannot be solely attributed to an increased presence of photorespiration in C_3_ species during fluctuating light, and the prevalence of this result across species from diverse evolutionary lineages and C_4_ subtypes suggests the ability sustain high photosynthetic rates after a transition to lower light may indeed be linked to other features of the C_4_ pathway—such as the large metabolite pools intrinsic to CCM operation (Lilley et al. 1977; Leegood and Furbank 1984; Stitt et al. 1985; Arrivault et al. 2017).

Metabolic pools also play a role in C_3_ species, and postillumination CO_2_ fixation has previously been attributed to altering pools of C_3_ intermediates and ATP and redox equivalents that accumulate during higher light fluctuations (Kaiser et al. 2015). In C_3_*A. semialata GMT*, C_3_*F. bidentis,* and C_3_*T. hassleriana*, the higher A_CO2_ values relative to steady state observed during 6 s fluctuations, as well as during the first half of the 30 s fluctuations (Fig. 3 BEH & KNQ) are in line with previous observations of carbon assimilation exceeding steady-state rates immediately after sunflecks (Pons and Pearcy 1992, Sharkey et al. 1986). However, metabolic pools in C_3_ species are typically considerably smaller than in C_4_ species (Borghi et al. 2022), which may be why C_4_*A. semialata MDG,* C_4_*F. bidentis*, and C_4_*G. gynandra* were able to sustain higher relative rates for longer than their C_3_ counterparts (Fig. 3), as evidenced by the higher average rates across longer fluctuations (Fig. 4 D-F). However, the capacity of C_4_ species to buffer through transitions to lower light still decreases across time as metabolite pools are depleted (Slattery et al. 2018), and carbon assimilation relative to steady-state during those periods was inversely related to the length of the fluctuations. Altogether, the higher and more sustained stimulation of C_4_ photosynthetic rates compared to C_3_ rates at low light is consistent with prior work (Laisk and Edwards 1997; Li et al. 2021; Lee et al. 2022) but the impact of light step duration explains why studies using different fluctuating light regimes can yield contrasting estimates for the comparative advantage of C_4_ versus C_3_ photosynthesis.

Although the comparative benefit of C_4_ species was observed under both oxygen conditions, the decrease in low-light A_CO2_ of the C_3_ species under 21% O_2_ was exacerbated by PIBs due to photorespiratory lagging. Previous work has shown that under photorespiration-suppressing conditions, C_3_ tree seedlings experience greater carbon gain under sunflecks than uniform light due to a less pronounced PIB, which maximizes postillumination CO_2_ fixation (Leakey et al. 2002). This is consistent with our observation that under 2% oxygen, there was greater assimilation relative to steady-state in the C3 species during the PIB time window than under 21% oxygen, which supports the idea that the suppression of photorespiration may have a specific benefit to dynamic light environments in C_3_ species (Way and Pearcy 2012).

Finally, the quantum yield of photosynthesis provides another indication of the storage capacity of C_4_ metabolic pools. In C_4_ species ФCO_2_ during the 6 and 30 s fluctuations was consistently above the theoretical maximum (Ehleringer and Pearcy 1983; Ishikawa et al. 2016, Morales et al. 2018, Yin and Struik 2018; Yin and Struik 2021), indicating that the energetic equivalent to ∼0.022 to 0.091 CO_2_/photon under 6 s fluctuations and ∼0.005 to 0.021 CO_2_/photon under 30 s fluctuations was being supplied outside of the thylakoid light reactions. The comparison with the maximum theoretical limit rather than with steady state ФCO_2_ was used to protect our conclusions against measurement uncertainty. The results, therefore, provide a very conservative estimate of the extent of decoupled CO_2_ fixation in C_4_ species, which may have been sustained by redox equivalents from malate decarboxylation, with demands for ATP and NADH being buffered through reversible reactions linking 3-PGA and TP, or interconversion of 3-PGA and PEP (Bräutigam et al. 2008; Stitt and Zhu 2014; Wang et al. 2014b; Arrivault et al. 2017; Slattery et al. 2018). Leaf-level and canopy simulations emphasize ФCO_2_ as the largest determinant of photosynthesis in the lower canopy (Gu et al. 2014; Bellasio and Farquhar 2019) and the stimulation of low light ФCO_2_ as observed here at 3 contrasting fluctuation frequencies could provide an important mitigation mechanism of the lower photosynthetic efficiency of C_4_ plants under low light (Ubierna et al. 2011; Medeiros et al. 2022).

### The C_4_ response during the transition to higher light could be related to the specific subtype metabolism

The comparative high-light performance of C_4_ photosynthesis was not uniform across the 3 genera. Although the use of a single representative example from each C_4_ subtype precludes the separation of species and subtype effects, the specific characteristics of the C_4_ pathway within each genus provide a possible explanation for the observed differences.

The highest A_CO2_ relative to steady state rates in C_4_ species during the 800 *µ*mol m^−2^ s^−1^ PFD periods were found in C_4_*A. semialata MDG* which is suggested to rely on a mixed NADP-ME-PEPCK C_4_ pathway (Ueno and Sentoku 2006) (Fig. 4 A–C). The lack of effect of light step duration on A_CO2_ during the higher light periods in *Alloteropsis* (Table 3) could be explained by photosynthetic induction during these fluctuating light regimes being relatively fast (Fig. 2 A–C). In contrast, high-light A_CO2_ relative to steady state was significantly lower with shorter fluctuations in both *Cleome* species; whereas in *Flaveria,* C_4_*F. bidentis* relative assimilation was more significantly reduced during shorter fluctuations than in C_3_*F. cronquistii*, indicating the C_4_ cycle lagged behind C_3_ activation (Table 3). The effect of fluctuation length on light induction in *Flaveria* and *Cleome* could be due to shorter fluctuations hindering the formation of metabolite pools necessary for optimal CCM operation, which has been suggested to result in impaired suppression of photorespiration and lagging photosynthetic induction during metabolite buildup (Sage and McKown 2006). Indeed, we previously found that the C_4_ species analyzed here were slower to induce photosynthesis from darkness relative to their C_3_ counterparts (Arce Cubas et al. 2023). Consistent with the faster induction observed in *Alloteropsis*, theoretical work indicates that not all subtypes are equally reliant on gradients: mixed C_4_ pathways like NADP-ME-PEPCK do not need metabolite gradients as large as NADP-ME or NAD-ME subtypes, as mixed subtypes can concurrently use different transfer acids (Wang et al. 2014a). The use of both Mal and Asp shuttles also allows for finer regulation of the ATP:NADPH ratio in response to changes in light, as only Mal transport brings redox equivalents into the BS (Yin and Struik 2021).

The suggested effect of subtype detailed above can also be observed in previous work. Li et al. (2021) compared a selection of 6 C_4_ species, 5 of which were NADP-ME and 1 NAD-ME, with 8 C_3_ species, concluding that C_4_ species utilized fluctuating light less efficiently by comparing obtained carbon assimilation during fluctuations to constant light values due to slower light induction. However, the role of species variation should not be underestimated—Lee et al. (2022) recently reported distinct species-specific induction patterns across 6 C_4_ grass species, which could also explain the results in this study, although similar induction patterns were still observable across NADP-ME species. The varying impact of light step duration on A_CO2_ found in this study also provides an important consideration for the interpretation of previous work. For example, Lee et al. concluded that their selection of C_4_ species assimilated more carbon under fluctuating light relative to steady state in comparison with 6 C_3_ species, seemingly in contrast with the conclusions by Li et al. (2021). However, although both experiments had low light steps of 2 min, high-light steps were 2 min in the Li et al. (2021) study and 4 min in Lee et al. (2022). Since both studies observed a slower decrease in photosynthetic rates relative to steady state values in C_4_ species after the transition to lower light, the additional 2 min of higher light in the Lee et al. study may have reduced the comparative penalty of C_4_ induction relative to the benefits of higher assimilation during the lower light periods, explaining the contrasting conclusions. Overall, this suggests C_4_ photosynthesis may have an advantage during brief periods of shade that intermit longer periods of sunlight commonly found in the top and middle layers of a leaf canopy (Kaiser et al. 2018). The comparative C4 advantage during “shade flecks” would allow for greater assimilation during high-light periods and maximize postillumination CO_2_ assimilation, despite the fact that the specific induction response may differ between C_4_ species. The comparative advantage of C_4_ photosynthesis after a transition to low light may be less impactful in shade environments interspersed with sunflecks like forest understories (Pearcy 1990), where carbon assimilation at low-light steady state dominates and the contribution of postillumination CO_2_ assimilation is less substantial.

### Fluctuations in light cause CO_2_ bursts in C_4_ G. gynandra

C_4_*G. gynandra* had distinctive assimilation kinetics after each light transition in the 300 s fluctuations (Fig. 2 I&R). At the start of the 800 *µ*mol m^−2^ s^−1^ PFD period, a rapid increase in A_CO2,_ described in previous work as a CO_2_ gulp (Laisk and Edwards 1997), was immediately followed by a CO_2_ burst, with another CO_2_ burst upon changing to the 100 *µ*mol m^−2^ s^−1^ PFD period. These bursts were not a product of photorespiration, as unlike the PIB observed after a transition to lower light in C_3_ species, in C_4_*G. gynandra* they occurred independent of oxygen concentration.

Previous studies on sunflecks (Laisk and Edwards 1997) have characterized the postillumination CO_2_ burst as a specific feature of the NAD-ME and PEPCK pathways, and other fluctuating light studies have also reported it primarily for NAD-ME species (Lee et al. 2022). Unlike NADP-ME species, where malate decarboxylation is linked to reducing equivalents from the C_3_ cycle, in NAD-ME species the C_3_ and C_4_ cycles are less tightly coupled—oxaloacetate is first reduced to malate and then decarboxylated in the mitochondria, but the redox balance is uncoupled from the C_3_ cycle (Ishikawa et al. 2016). This can result in excess CO_2_ being released despite insufficient RuBP regeneration upon a transition to lower light, causing unfixed CO_2_ to leak out of the BS and A_CO2_ to drop.

The CO_2_ gulp and burst at the start of the 800 *µ*mol m^−2^ s^−1^ PFD period is similar to induction kinetics observed in short dark-light fluctuations in NAD-ME *Amaranthus cruentus* (Laisk and Edwards 1997). These were attributed to the formation of alanine from the decarboxylation of aspartate in low light (or darkness), leading to rapid conversion of alanine to pyruvate followed by phosphorylation to PEP when light is increased (Laisk and Edwards 1997; Lee et al. 2022). The initial PEP carboxylation following the increase in light exceeds the rate at which PEP pools can be replenished but subsequently crashes and readjusts while PEP regeneration is re-established. This may account for the observed temporary gulp and subsequent steady increase in A_CO2_. Furthermore, the centripetal chloroplast positioning found in NAD-ME species (Yoshimura et al. 2004) could increase the path length for metabolites and CO_2_, and lead to a more pronounced form of the biphasic induction previously observed in NADP-ME species and attributed to C_4_ cycle limitations (Lee et al. 2022).

## Conclusions

The presented work compared C_4_ to C_3_ photosynthesis in response to fluctuating light. By using 3 independent phylogenetically controlled comparisons and fluctuations with 3 contrasting light step durations the presented work circumvented issues in previous studies to yield more robust conclusions. The results showed that the stimulation of A_CO2_ in the low-light phase was both higher and more sustained in C_4_ photosynthesis across all 3 comparisons, suggesting this could be a common comparative advantage of C_4_ photosynthesis. In contrast, observed patterns of A_CO2_ in the high-light phase were found to be more variable across genera rather than attributable to photosynthetic pathway, which could potentially be related to the specific C_4_ subtype.

## Materials and methods

### Plant materials

To control for evolutionary distance, 3 pairs of phylogenetically linked *Alloteropsis*, *Flaveria*, and *Cleome* C_3_ and C_4_ species (shown in Table 5, see also Arce Cubas et al. 2023) were selected. Substantial evolutionary distance exists between the selected genera and C_4_ photosynthesis evolved independently in each. The C_4_ origin dates back approximately 17 million years (Ma) in *Cleome*, ∼ 2 Ma in *Flaveria*, and is even more recent in *Alloteropsis* (Christin et al. 2011; Lundgren et al. 2015). In addition, the selected species include both monocots (*Alloteropsis*) and dicots (*Flaveria* and *Cleome*), and all 3 major decarboxylase enzymes of the C_4_ pathway: NADP-ME-PEPCK (C_4_*Alloteropsis semialata MDG*), NAD-ME (C_4_*Flaveria bidentis*), and NAD-ME (C_4_*Gynandropsis gynandra*) (Ueno and Sentoku 2006; Bräutigam et al. 2008; Gowik et al. 2011).

**Table 5.** Phylogenetically linked C_3_ and C_4_*Alloteropsis*, *Flaveria*, and *Cleome* species used in this study with their photosynthetic pathway, class, and main C_4_ subtype if applicable

Genus	Species and accession	Photosynthetic pathway	C4 subtype	Class
*Alloteropsis*	*Alloteropsis semialata* subspecies *semialata* accession GMT	C_3_	—	Monocot
	*Alloteropsis semialata* subspecies *eckloniana* accession MDG	C_4_	NADP-ME-PEPCK	
*Flaveria*	*Flaveria cronquistii*	C_3_	—	Dicot
	*Flaveria bidentis*	C_4_	NADP-ME	
*Cleome*	*Tarenaya hassleriana*	C_3_	—	
	*Gynandropsis gynandra*	C_4_	NAD-ME	

### Plant growth and propagation

*Flaveria* and *Cleome* species were grown in a Conviron walk-in growth room (Conviron Ltd., Winnipeg, MB, CA) at 20°C, 60% relative humidity (RH), and 150 *µ*mol m^−2^ s^−1^ PFD over a 16-h photoperiod; and the *Alloteropsis* accessions in a glasshouse in Cambridge, England, at 18 to 25°C, 40% to 60% RH, with supplemental lighting to provide a minimum of 600 to 700 *µ*mol m^−2^ s^−1^ PFD over a 16-h photoperiod in addition to incoming irradiance. All plants were well-watered and grown in Levington Advance M3 compost (Scotts, Ipswich, UK) mixed with Miracle-Gro All Purpose Continuous Release Osmocote (Scotts Miracle-Gro Company, Marysville, OH, USA; 4 L compost: 25 g Osmocote), with vermiculite being added to the *Alloteropsis* soil mix to prevent waterlogging (1 L vermiculite: 4 L compost: 25 g Osmocote).

*Alloteropsis* and *Flaveria* species were vegetatively propagated whilst *Cleome* species were grown from seed. *Alloteropsis* MDG and GMT accession tillers were grown in 2 L pots and all gas exchange measurements were taken after 2 weeks. For *Flaveria* propagation, lateral shoot cuttings were dipped in Doff Hormone Rooting Powder (Doff Portland Ltd., Hucknall, UK) to induce root development, grown on 0.25 L pots, and measured after 8 to 10 weeks. *Flaveria cronquistii* requires vegetative propagation, so *F. bidentis* plants were first grown from seed and subsequently propagated via cuttings*. Cleome* germination was induced with a 30°C/20°C day/night cycle for *Tarenaya hassleriana*, and at 30°C for *G. gynandra*. The germinated seeds were sown in 24-cell trays before transfer to 0.25 L pots. Due to the different developmental rates of the *Cleome* species, germination was staggered so both species could be measured at approximately the same developmental stage, after 8 to 10 weeks for *G. gynandra* and 4 to 6 weeks for *T. hassleriana*. All plants were measured during vegetative state.

### Gas exchange measurements at 21% and 2% O_2_

Gas exchange under steady and fluctuating light conditions was measured on young, fully expanded leaves using an open gas exchange system (LI-6800, LI-COR, Lincoln, NE, USA) with a Multiphase Flash Fluorometer (MPF) chamber (6800-01A, LI-COR). Chamber conditions were controlled at 410 ppm sample CO_2_ concentration, 60% relative humidity with average leaf vapour pressure deficit (VPD) of 1.3 ± 0.1 kPa, 25°C heat exchanger temperature, and flow rate of 600 *µ*mol s^−1^. Actinic light was provided by the MPF and composed of 90% red (625 nm) and 10% blue light (475 nm).

For experiments in 2% O_2_, a pre-mixed 2% O_2_ and 98% N_2_ gas mixture (BOC Ltd., Woking, UK) was supplied to the LI-6800 through the air inlet using a mass flow controller (EL-FLOW, Bronkhorst Hight-tech BV, Ruurlo, NL) and an open T-junction to regulate constant surplus flow according to manufacturer instructions. The LI-6800 Infrared Gas Analyser (IRGA) calibration was adjusted to the O_2_ concentration in the instrument constants before measurement.

### Steady state light response curves

Photosynthetic responses to steady light were measured for all species at both 21% and 2% O_2_. Leaves were illuminated with 1000 *µ*mol m^−2^ s^−1^ PFD for 20 to 40 min to allow CO_2_ assimilation and stomatal conductance to reach steady state, and gas exchange was subsequently measured in a descending gradient of light intensity: 2000, 1700, 1500, 1200, 1000, 800, 600, 400, 300, 200, 100, 75, 30, and 0 *µ*mol m^−2^ s^−1^ PFD. Gas exchange parameters were logged between 120 and 240 s at a given light intensity, when leaf intracellular CO_2_ concentration (C_i_), and CO_2_ assimilation (*A_CO2_*) were stable.

Respiration in the light (*Rd*) was estimated from the y-intercept of a linear regression of the slope before the inflection point. Measurements at 0 and 30 *µ*mol m^−2^ s^−1^ PFD were not included in the regression to account for the Kok effect (Kok 1949). Photosynthetic rates and C_i_ at 100 and 800 *µ*mol m^−2^ s^−1^ PFD were taken as the steady state values for comparison with fluctuating light measurements. The quantum yield of CO_2_ assimilation (ΦCO_2_) was calculated from net CO_2_ assimilation (A_CO2_), absorbed PFD (PFD_abs_), and *Rd* using Equation (1):


(1) $$\phi CO_{2}=\frac{A_{CO2}+Rd}{PFD_{abs}}\;$$

Fluctuating light experiments, correction for dynamic conditions, and analysis

To measure photosynthetic responses to fluctuating light, leaves were first acclimated at 150 *µ*mol m^−2^ s^−1^ PFD, the minimum growth light intensity of all species, for 30 to 60 min until stomatal conductance and photosynthetic rates reached constant levels. Using a custom program, leaves were then exposed to repetitive stepwise fluctuations in light intensity from 800 to 100 *µ*mol m^−2^ s^−1^ PFD for 1 h, with gas exchange parameters recorded every 2 s. Three different light treatments were tested, with each light step lasting 6, 30, or 300 s. To avoid interference with the shorter fluctuations and the data sampling interval, the averaging time of the LI-6800 logging was kept minimal (averaging time was set to 0), meaning that each log should represent an average of the preceding 0.5 s, the inverse of the instrument digital update frequency of 2 Hz. The IRGAs were only matched before the program starting. Experiments were conducted at both 21% and 2% O_2_ and the light treatments and oxygen concentration were randomized within each phylogenetic pair.

As measurements during light fluctuations violate the steady state assumption underlying default rate equations, a storage flux correction was applied that follows the same principle as the dynamic assimilation technique previously developed for fast CO_2_ and light response curves (Saathoff and Welles 2021). Saathoff and Welles (2021) show that based on the mass balance of the instrument cuvette, the derivative of the cuvette concentration over time can be used to adjust carbon assimilation and transpiration rates. Accordingly, here Equations (2) and (3) were used to compute derivatives from the time-series data and adjust the steady state *E* and A_CO2_ rates– and other instrument calculations derived from these


(2) $$Storage\;flux_{H2O}=\frac{\frac{PV}{RT}x\Delta H_{2}O\;}{S\;x\;t},$$

(3) $$Storage\;flux_{CO2}=\frac{-\frac{PV}{RT}x\Delta CO_{2}\;}{S\;x\;t}.$$

The equations use the ideal gas law, where *P* represents pressure (Pa, from instrument recordings), *V* represents cuvette volume (8.67e^−5^ m^3^), *R* represents the molar gas constant, and *T* represents temperature to calculate the change in moles of gas of CO_2_ or H_2_O using instrument recordings of current and previous gas concentration. *S* represents leaf area (m^−2^) and *t* represents the time since last log (s) and are used to convert the molar concentrations to flux per area. The signum reconciles CO_2_ flux with scientific convention for assimilation.

### Leaf absorptance

After gas exchange measurements, the spectral qualities of the leaves were measured with an integrating sphere (LI-1800-12, LI-COR) optically connected to a miniature spectrometer (STS-VIS, Ocean Insight, Orlando, FL, USA) following manufacturer instructions (LI-COR 1988). Leaf absorptance (*L_abs_*) was calculated using Equation (4), where *T_s_* and *R_s_* are transmittance and reflectance of a diffuse sample


(4) $$L_{abs}=1-T_{s}-R_{s}.$$

For the light response curves, incident PFD was converted to absorbed PFD using L_abs_ of the red and blue emission wavelengths of the 6800-01A MPF light source. For the specific absorptance values, see Supplemental Table S5.

### Data processing

Data from the last 10 min of the fluctuating light treatment were used for analysis to ensure the effects observed were due to fluctuations and not induction, which was apparent during the first 30 min of the time series (Supplemental Fig. S1). To analyze the relative performance of each species, net photosynthesis (A_CO2_) under fluctuating light was expressed as a percentage of the steady state rates achieved at the corresponding light intensity. Additionally, ΦCO_2_ under fluctuating light was calculated using Equation (1).

The area under the curve (AUC) (Makowski et al. 2019) of A_CO2_ relative to steady state and ΦCO_2_ during the 100 and 800 *µ*mol m^−2^ s^−1^ PFD periods of the light treatment was integrated using the trapezoidal rule (Jawień 2014) and divided by the duration to obtain an average value for each light level which was used to compare between fluctuations of different length and, in the case of Φ_CO2_, directly to steady-state values. To compare differences in PIB between 21% and 2% oxygen, the analysis of A_CO2_ relative to steady state was also performed specifically during the time of the 100 *µ*mol m^−2^ s^−1^ PFD periods where the PIB was evident: 10 to 30 s in the 30 s fluctuations, and 10 to 70 s in the 300 s fluctuations. The PIB was not observed during the initial 10 s, hence the 6 s fluctuations were not included in this analysis.

### Statistical analysis

Each phylogenetically controlled comparison was run as an independent experiment, thus statistical analyses were conducted separately on paired *Alloteropsis*, *Flaveria,* and *Cleome* light response curves (*800* A_CO2_, *100* A_CO2_, C_i 800_, C_i 100_, *800* ΦCO_2_, *100* ΦCO_2_, *Rd*), and fluctuating light measurements (A_CO2_ relative to steady state, ΦCO_2_). Two-way ANOVA was used to test for the effects of photosynthetic pathway, oxygen concentration, and their interactions on steady-state photosynthesis parameters; and three-way ANOVA to test for the effects of photosynthetic pathway, fluctuation length, oxygen concentration, and their interactions on A_CO2_ relative to steady state and ΦCO_2_. Specifically for the ΦCO_2_ analysis, the quantum yields obtained under steady state at 800 and 100 *µ*mol m^−2^ s^−1^ PFD were included in the dataset as an additional fluctuation length. For each ANOVA, assumptions of normality, homogeneity of variance, and sphericity were satisfied. Mean and standard error of the mean for steady-state photosynthesis parameters, A_CO2_ relative to steady state, and ΦCO_2_ across the light fluctuation regimes were calculated for reporting.

All data analysis and plot generation was done with R 4.1.1 (R Core Team 2021) on RStudio 2022.12.0 + 353 (Posit Team 2022) using the tidyverse (Wickham et al. 2019), RColorBrewer (Neuwirth 2014), lme4 (Bates et al. 2015), and bayestestR libraries (Makowski et al. 2019).

## Supplementary Material

kiad355_Supplementary_Data

## Data Availability

Data available upon request to the corresponding author.

## Supplemental data

The following materials are available in the online version of this article.

**Supplemental Figure S1.** Net CO_2_ assimilation (A_CO2_) in phylogenetically linked C_3_ and C_4_*Alloteropsis*, *Flaveria*, and *Cleome* species under 3 different fluctuating light regimes at 21% and 2% O_2_.

**Supplemental Figure S2.** Net CO_2_ assimilation (A_CO2_) across the 800 and 100 *µ*mol m^−2^ s^−1^ PFD light steps.

**Supplemental Figure S3.** Net CO_2_ assimilation (A_CO2_) relative to steady state (%) across the 800 PFD light steps only.

**Supplemental Figure S4.** Boxplots of net carbon assimilation (A_CO2_) under the 800 and 100 *µ*mol m^−2^ s^−1^ PFD periods of the fluctuating light regimes or from steady-state measurements.

**Supplemental Table S1.** Leaf absorptance values of phylogenetically linked C_3_ and C_4_*Alloteropsis*, *Flaveria*, and *Cleome* species.
