# Supplementary material for: Lessons from relatives: C4 photosynthesis enhances CO2 assimilation during the low-light phase of fluctuations
Source: Plant Physiol. 2023 Jun 19;193(2):1073–90. doi: 10.1093/plphys/kiad355 (PMC10517189; doi:10.1093/plphys/kiad355)
Supplement: kiad355_Supplementary_Data [file kiad355_supplementary_data.pdf]

# Supplemental Data

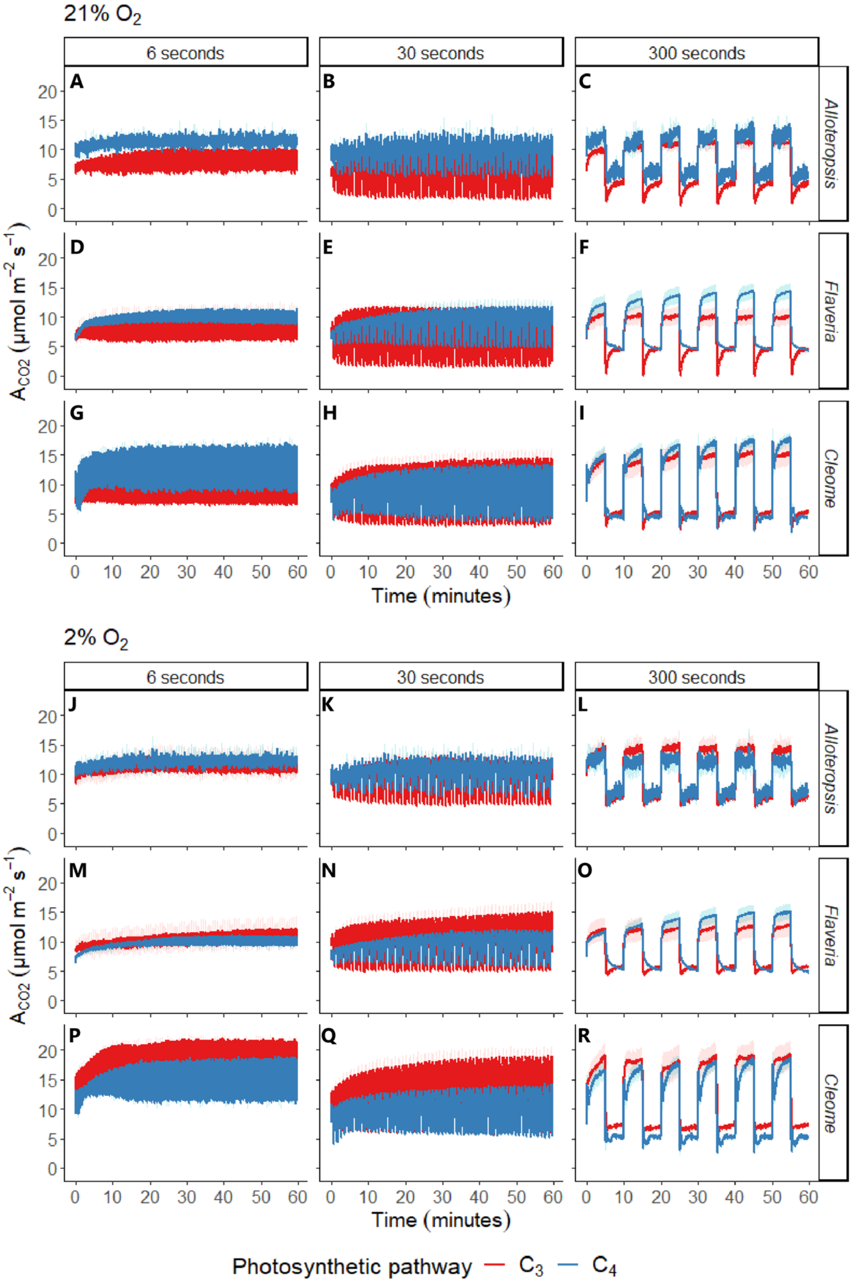


**Supplemental Figure S1:** Net $CO_2$ assimilation ($A_{CO2}$) in phylogenetically linked $C_3$ and $C_4$ *Alloteropsis*, *Flaveria* and *Cleome* species under three different fluctuating light regimes at 21% (A-I) and 2% $O_2$ (J-R). Each light regime consisted of alternating 800 and 100 µmol $m^{-2}$ $s^{-1}$ PFD periods, where each light step lasted 6, 30, or 300 seconds before changing. Treatments were started after leaves were acclimated at 150 µmol $m^{-2}$ $s^{-1}$ PFD and lasted 1 hour, but data in the study was taken from minutes 50-60 of each treatment. Ribbons represent standard error of the mean (n=5).

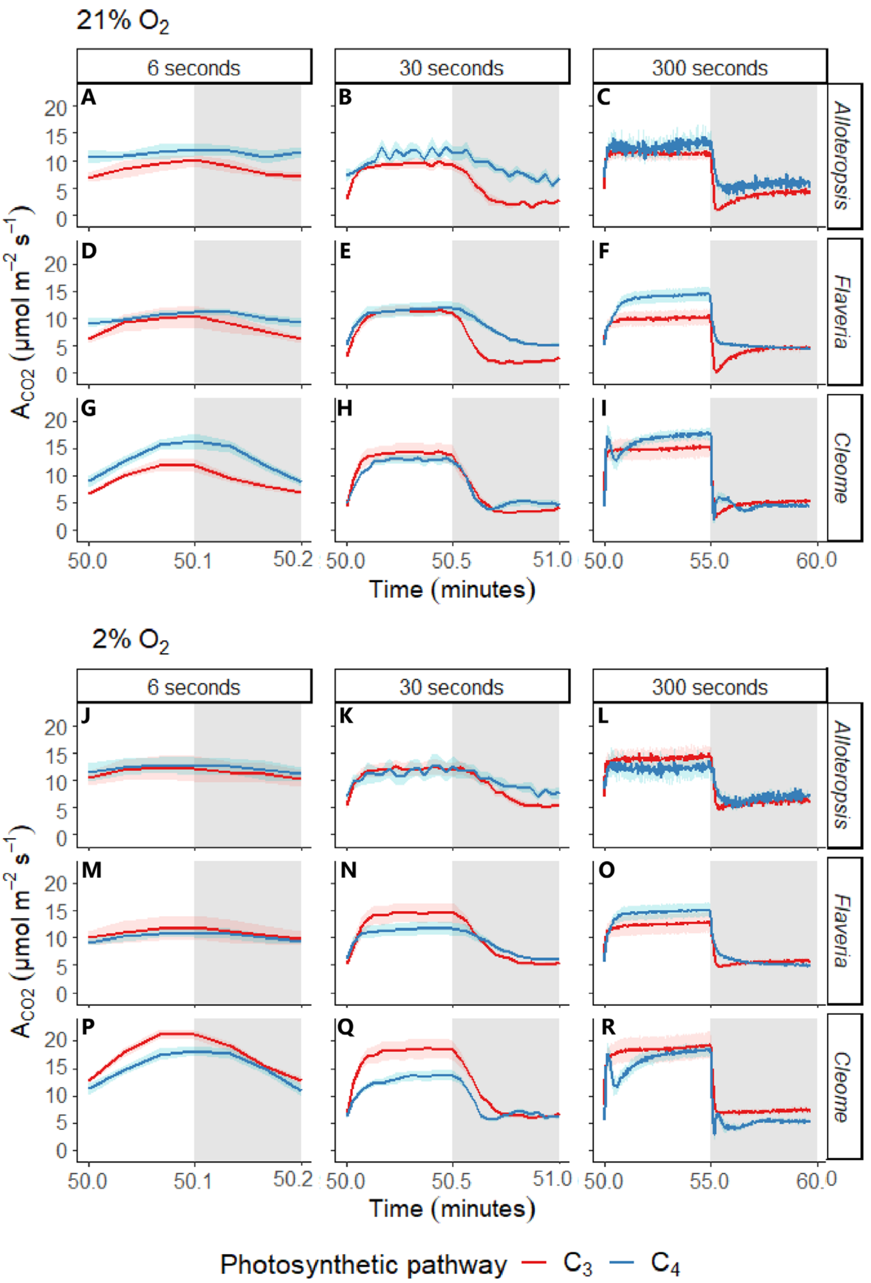


**Supplemental Figure S2:** Net $CO_2$ assimilation ($A_{CO2}$) across the 800 and 100 µmol $m^{-2}$ $s^{-1}$ PFD light steps, in white and grey respectively. All data was taken starting from 50 min into the experiment. Depending on the fluctuating light treatment, each light step was 6, 30, or 300 seconds. Values represent phylogenetically linked $C_3$ and $C_4$ *Alloteropsis*, *Flaveria* and *Cleome* species at 21% (A-I) and 2% $O_2$ (J-R). Ribbons represent standard error of the mean (n=5). The corresponding $A_{CO2}$ relative to steady state (%) values are in **Figure 4**.

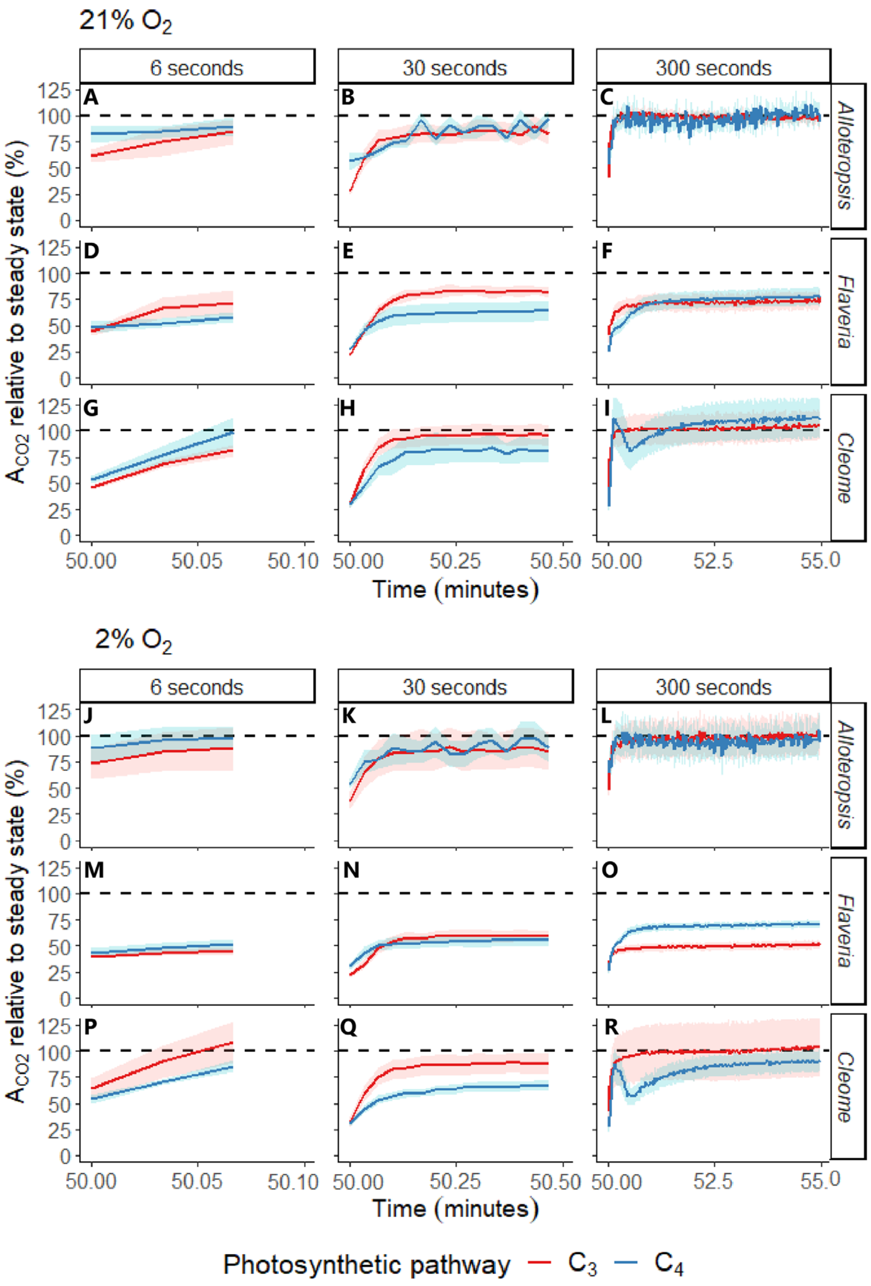


**Supplemental figure 3:** Net $CO_2$ assimilation ($A_{CO2}$) relative to steady state (%) across the 800 PFD light steps only for better resolution, starting at the 50 minute mark. Depending on the fluctuating light treatment, subplots are showing a higher light phase of 6, 30, or 300 seconds. Values represent $A_{CO2}$ at a given point in the fluctuating light treatment relative to $A_{CO2}$ obtained from steady state light response curves at the light intensity of each period in phylogenetically linked $C_3$ and $C_4$ *Alloteropsis*, *Flaveria* and *Cleome* species at 21% (A-I) and 2% $O_2$ (J-R). The dashed line represents 100%, where assimilation would be exactly that of steady state. Ribbons represent standard error of the mean (n=5).

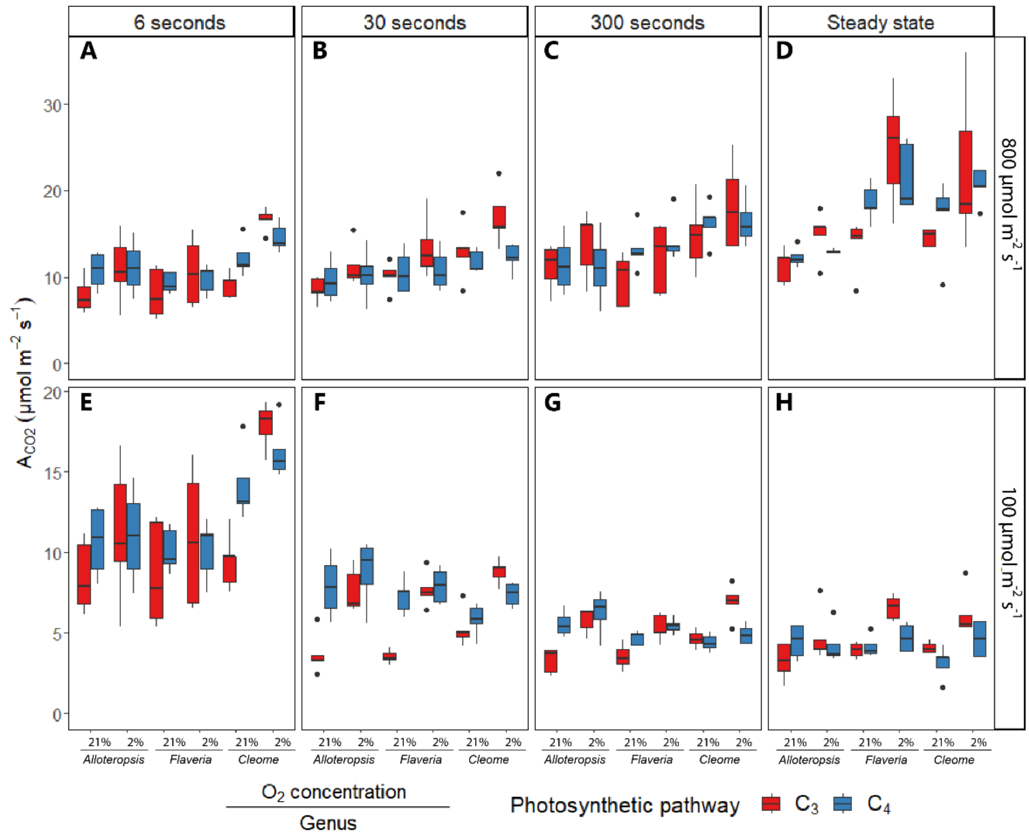


**Supplemental figure 4:** Boxplots of net carbon assimilation ($A_{CO2}$) under the 800 and 100 µmol m$^{-2}$ s$^{-1}$ PFD periods of the fluctuating light regimes. Each regime consisted of alternating 800 (A-D) and 100 µmol m$^{-2}$ s$^{-1}$ PFD (E-H) periods, where each light step lasted 6, 30, or 300 seconds before changing. The plot includes $A_{CO2}$ steady state values taken from the light response curves. The area under the curve (AUC) $A_{CO2}$ of for each period across the timeseries for phylogenetically linked $C_3$ and $C_4$ *Alloteropsis*, *Flaveria* and *Cleome* species at 21% or 2% $O_2$ was calculated from between minutes 50-60 of each fluctuating light treatment and converted to a rate for ease of comparison. Box edges represent the lower and upper quartiles, the solid line indicates the median, and points represent outliers beyond 1.5 times the interquartile range (n = 5 for each combination of species/measurement condition).

**Supplemental Table 1:** Leaf absorptance values of phylogenetically linked $C_3$ and $C_4$ *Alloteropsis*, *Flaveria*, and *Cleome* species from the blue (475 nm) and red (625 nm) wavelengths of the actinic light source used in experiments, measured with an integrating sphere. Means and standard error of the mean are shown (n = 5).

| **Genus** | **Species** | **$L_{abs}$ (475 nm)** | **$L_{abs}$ (625 nm)** |
|---|---|---|---|
| *Alloteropsis* | C3 *A. semialata GMT* | 0.90±0.02 | 0.88±0.02 |
| | C4 *A. semialata MDG* | 0.87±0.02 | 0.81±0.02 |
| *Flaveria* | C3 *F. cronquistii* | 0.88±0.02 | 0.85±0.02 |
| | C4 *F. bidentis* | 0.94±0.00 | 0.90±0.00 |
| *Cleome* | C3 *T. hassleriana* | 0.95±0.00 | 0.92±0.00 |
| | C4 *G. gynandra* | 0.93±0.00 | 0.89±0.00 |
